# Altered iron and myelin in premanifest Huntington's Disease more than 20 years before clinical onset: Evidence from the cross-sectional HD Young Adult Study

**DOI:** 10.1016/j.ebiom.2021.103266

**Published:** 2021-03-09

**Authors:** Eileanoir B. Johnson, Christopher S. Parker, Rachael I. Scahill, Sarah Gregory, Marina Papoutsi, Paul Zeun, Katherine Osborne-Crowley, Jessica Lowe, Akshay Nair, Carlos Estevez-Fraga, Kate Fayer, Geraint Rees, Hui Zhang, Sarah J. Tabrizi, the HD-YAS Investigators

**Affiliations:** a Huntington's Disease Centre, Department of Neurodegenerative disease, UCL Queen Square Institute of Neurology, University College London, London, UK; b Centre for Medical Image Computing, Department of Computer Science, UCL, London, UK; c IXICO Plc, London, , UK; d Division of Equity, Diversity and Inclusion, University of New South Wales, Sydney, New South Wales, Australia; e Max Planck University College London Centre for Computational Psychiatry and Ageing Research, UCL Queen Square Institute of Neurology, London, UK; f University College London Institute of Cognitive Neuroscience, University College London, London, UK; g Dementia Research Institute at University College London, London, UK

**Keywords:** Huntington's disease, MRI, Microstructure, Iron, Myelin

## Abstract

**Background:**

Pathological processes in Huntington's disease (HD) begin many years prior to symptom onset. Recently we demonstrated that in a premanifest cohort approximately 24 years from predicted disease onset, despite intact function, there was evidence of subtle neurodegeneration. Here, we use novel imaging techniques to determine whether macro- and micro-structural changes can be detected across the whole-brain in the same cohort.

**Methods:**

62 premanifest HD (PreHD) and 61 controls from the HD Young Adult Study (HD-YAS) were included. Grey and white matter volume, diffusion weighted imaging (DWI) measures of white matter microstructure, multiparametric maps (MPM) estimating myelin and iron content from magnetization transfer (MT), proton density (PD), longitudinal relaxation (R1) and effective transverse relaxation (R2*), and myelin g-ratio were examined. Group differences between PreHD and controls were assessed; associations between all imaging metrics and disease burden and CSF neurofilament light (NfL) were also performed. Volumetric and MPM results were corrected at a cluster-wise value of familywise error (FWE) 0.05. Diffusion and g-ratio results were corrected via threshold-free cluster enhancement at FWE 0.05.

**Findings:**

We showed significantly increased R1 and R2*, suggestive of increased iron, in the putamen, globus pallidum and external capsule of PreHD participants. There was also a significant association between lower cortical R2*, suggestive of reduced myelin or iron, and higher CSF NfL in the frontal lobe and the parieto-occipital cortices. No other results were significant at corrected levels.

**Interpretation:**

Increased iron in subcortical structures and the surrounding white matter is a feature of very early PreHD. Furthermore, increases in CSF NfL were linked to microstructural changes in the posterior parietal-occipital cortex, a region previously shown to undergo some of the earliest cortical changes in HD. These findings suggest that disease related process are occurring in both subcortical and cortical regions more than 20 years from predicted disease onset.

**Research in context**

**Evidence before this study**

Huntington's Disease (HD) is a monogenic, neurodegenerative disease characterised by motor, cognitive and neuropsychiatric symptoms that appear long before clinical diagnosis. By investigating early premanifest HD (PreHD), the earliest stages of neurodegeneration can be characterised. Primarily, medium spiny projection neurons in the basal ganglia begin to degenerate early and as such, subcortical atrophy has been robustly identified in PreHD gene-carriers. HD pathogenesis, however, is not restricted to just macrostructural change and there is evidence of early microstructural abnormalities in white matter pathways around 15 years prior to disease onset, along with evidence of disrupted myelination and iron accumulation. The exact mechanisms of these changes are unclear, but neuronal loss may lead to remyelination of white matter fibres and a congruent increase of iron-rich oligodendrocytes to support this process. Alternatively, disrupted iron homeostasis could results in iron accumulation, associated with the presence of the Huntingtin gene. The interplay between neuronal death (as measured by brain volume) and microstructural changes to myelin and iron has yet to be investigated in early PreHD.

**Added value of this study**

Our recent study in young gene-positive adults, estimated to be on average 24 years from predicted onset, showed increased neurofilament light (NfL), a marker of axonal degeneration, along with significantly reduced putamen volume. Here, we have investigated whole-brain macro and microstructural properties associated with HD pathology in this cohort of asymptomatic PreHD gene-carriers. Specifically, grey and white matter volume, diffusion weighted imaging (DWI) measures of white matter microstructure, multiparametric maps (MPM) that estimate myelin and iron content, and a composite measure of diffusion and MPM data used to estimate g-ratio, a measure of axonal myelination, were quantified. While there were no differences in volume or white matter microstructure (either DWI or g-ratio), PreHD gene-carriers displayed significantly higher measurements in two MPM metrics - longitudinal relaxation (R1) and effective transverse relaxation (R2*) - in the putamen, globus pallidus and external capsule. The higher values are indicative of increased iron in these areas. For PreHD individuals, there was also an association between higher CSF NfL and lower R2* in the cortex; this finding suggests either reduced iron or reduced myelin in these regions is associated with increased axonal degeneration.

**Implications of all the available evidence**

In characterising microstructural changes in early PreHD gene-carriers, there is a suggestion that HD pathological processes begin more than 20 years from predicted onset. This not only has implications in terms of HD pathology, but also the wider field of neurodegenerative disease and the role of iron accumulation in the development neurodegenerative symptoms and their trajectory across the life course of disease. Furthermore, as potential disease-modifying treatments for neurodegeneration move into phase III clinical trials, identifying the earliest pathological changes is vital before therapies can be administered to PreHD gene-carriers.

Alt-text: Unlabelled box

## 1 Introduction

Huntington's disease (HD) is a monogenic neurodegenerative disorder with a definitive genetic test, and a long pre-manifest period that allows us to characterise the very earliest stages of neurodegeneration. Clinical symptom onset is driven by age and CAG repeat length in the huntingon gene [1], and validated models (e.g. Langbehn et al [2]) allow us to predict when premanifest gene carriers are likely to convert to manifest disease. As potential disease-modifying treatments for neurodegeneration move into phase III clinical trials, identifying the earliest pathological changes is vital before therapies can be administered to presymptomatic HD gene-carriers. MRI studies show that brain atrophy occurs up to 15 years before onset of symptoms [3,4] however the specific pathological origin of signal changes detected by T1 weighted structural MRI is difficult to establish [5]. Volumetric changes are likely to be preceded by subtle microstructural alterations, as indicated from studies of post-mortem HD brains and mouse-models of HD [6,7].

Both post-mortem studies and mouse-models of HD implicate cell death, disrupted myelin processes and iron accumulation in HD pathogenesis, but the exact timing and interplay between these processes, along with their impact on symptomatology, is not yet clear. Cell death was one of the earliest post-mortem observations described in HD [6] with medium spiny projection neurons (MSNs) in the basal ganglia particularly vulnerable to early degeneration, indicating that abnormal myelination processes may be involved in the pathogenesis of HD [7]. As MSNs degenerate, there is thought to be resultant re-myelination and thus an increase in the presence of iron-rich oligodendrocytes to support this process [8,9]. However, abnormal iron distribution and accumulation could also result from disrupted iron homeostasis, to which the Huntingtin protein (HTT) has been linked [10].

While post-mortem and mouse studies help to characterise HD at a microscopic level, neuroimaging offers the unique opportunity to study HD brain changes in real-time *in-vivo*. Numerous MRI studies have corroborated mouse and post-mortem work, reporting significant subcortical iron accumulation as a feature of HD pathology [7,[11], [12], [13], [14]], with increasing iron associated with decreasing subcortical volume [14], [15], [16], [17]. In addition, microstructural imaging techniques such as diffusion weighted imaging (DWI) have provided significant evidence that disruptions in white matter (WM) microstructure are detectable at least 14 years before onset [18]. While it is clear that changes in volume, WM microstructure and iron can be detected via MRI in PreHD, it is imperative that the earliest point at which microstructural changes can be detected in HD is identified.

Recently, we found evidence that despite intact function, a group of far from onset HD gene-carriers show subtle signs of a decline in neuronal health. Our HD Young Adult Study (HD-YAS) cohort [19] consists of 64 HD gene-carriers approximately 24 years from predicted disease onset. Compared to 67 age, sex and education matched controls, they showed significantly increased neurofilament light (NfL), a marker of axonal degeneration, along with significantly reduced putamen volume. Here, we extend this work using whole-brain approaches, undertaking an exploratory analysis to elucidate whether there are any detectable early pathological processes occurring beyond the caudate, putamen and surrounding white matter in this group. Our previous analysis was performed on carefully selected regions-of-interest, but we hypothesise that there are early changes occurring beyond these regions which can be detected with a whole-brain analysis.

To enable us to detect the earliest microstructural changes in PreHD we use multiparametric maps (MPMs) and multi-shell DWI. These techniques allow us to examine a range of different micro-structural properties of the tissue. MPM is one technique that aims to probe specific microstructural tissue properties such as iron and myelin via the use of multiple contrast parameters [20]. Typically, four quantitative measures are derived from MPMs, with each measure having differential sensitivity to underlying biological metrics which reflect iron and myelin properties. Magnetic transfer saturation (MT) measures macromolecular content, particularly myelin so that a decrease in MT represents demyelination. Proton density (PD) maps are most sensitive to microstructural water content, while longitudinal relaxation rate (R1) detects the relative contribution of myelin and water content as well as paramagnetic content such as iron, and effective transverse relaxation rate (R2*) is most sensitive to iron and myelin distribution. The results from each MPM measure are interpreted in the context of each other and the regions studied, for example changes in R1 or R2* in subcortical structures with low myelin content are suggestive of iron changes, whereas changes in cortical or white matter may be due to iron or myelin content. MPMs can also be combined with DWI data to estimate g-ratio [21], a measure of axonal myelination thought to reflect the ratio of the inner- to outer- myelin sheath diameter.

We seek to establish whether disease-related micro- or macro- structural changes can be detected more than 20 years prior to predicted HD onset using a range of complementary state-of-the-art whole-brain imaging methods. Our unique cohort of 64 PreHD participants predicted to be 24 years from disease onset, and 67 matched controls underwent the following multi-modal imaging assessments: T1-weighted images were analysed via voxel-based morphometry (VBM) to measure brain volume; Multi-shell DWI acquisition with tract-based spatial statistics (TBSS) was used to investigate WM microstructure; Multiparametric maps (MPMs) were analysed to quantify MT, PD, R1 and R2* in an investigation of iron and myelin. Finally, we combine the DWI and MPM data to estimate the g-ratio and compare this between control and PreHD groups with TBSS. We then undertake whole-brain voxel-wise correlation analyses to determine whether any MRI measures are associated with disease burden or CSF neurofilament light (NfL).

## 2 Methods

### 2.1 Participants

Participants were recruited as part of the HD young adult study (HD-YAS [19]); a single-site observational study of 64 PreHD and 67 control participants, aged between 18 and 40, matched for age, sex and education. Participants were recruited through HD and genetics clinics across the UK and patient support groups, with visits conducted at the London Hospital for Neurology and Neurosurgery between 2^nd^ August 2017 and 25^th^ April 2019. The study was originally powered for primary hypothesis testing of striatal volume differences with 80% power and 5% type 1 error to detect group differences of 0.53 adjusted within-group standard deviations.

PreHD participants were required to have had a positive genetic test for HD with a cytosine, adenine, guanine (CAG) repeat >39 confirming that they will develop HD, whilst also having a Disease Burden Score (DBS) < 240 [22] and a Unified Huntington's Disease Rating Scale Diagnostic Confidence Score (UHDRS DCS) < 4 confirming premanifest status [23]. DBS is an estimated exposure to the mutant huntingtin protein and thus approximates disease stage, and is calculated as (CAG – 35.5) x current age. PreHD participants were only recruited if they had no signs or symptoms of HD. Estimated time to onset was calculated using the Langbehn formula [2]. Control participants were gene-negative, HD family members or individuals with no familial history of HD. Exclusions at screening included drug or alcohol abuse and/or dependence, neurological or psychiatric co-morbidity or contraindication to MRI. As part of the study, all participants underwent an examination of clinical and medical history, along with an extensive cognitive and neuropsychiatric battery, a neuroimaging session, blood sampling and optional cerebrospinal fluid collection.

The study was approved by the London Authority Bloomsbury Research Ethics Committee (Ref no: 16/LO/1323) and all participants gave written informed consent prior to study entry. See [19] for full recruitment criteria and study procedures.

### 2.2 Imaging acquisitions

MRI data were acquired on a 3T Prisma Scanner (Siemens Healthcare, Germany) with a 64 channel head coil. T1-weighted images (T1w) were acquired with a 3D MPRAGE sequence: TR=2530ms; TE=3.34ms; TI=1100ms; flip angle=7°; FOV=256 × 256 × 176mm^3^ with a resolution of 1.0 × 1.0 × 1.0 mm^3^ with a total scan time 6 minutes 3 seconds. Multi-shell diffusion-weighted images (DWI) were acquired with a spin-echo echo-planar imaging (EPI) sequence: EPI factor 110; TR=3260ms; TE=58ms; 72 slices with slice thickness 2mm; in-plane FOV 220 × 220 mm^2^ with a resolution 2.0 × 2.0 mm^2^; b-values=0 (n=10), 100 (n=8), 300 (n=8), 1000 (n=64) and 2000 (n=64) s/mm^2^; multi-band acceleration factor 2 with time-shifted RF pulses; a total acquisition time of 12 minutes. One additional b=0 volume was acquired with reverse (posterior to anterior direction) phase encoding (PE) and all other volumes with forward PE.

The Multiparametric mapping (MPM) acquisition protocol consisted of three differently weighted 3D multi-echo FLASH acquisitions: quantitative Magnetisation Transfer weighted (MTw), quantitative Proton Density weighted (PDw) and quantitative T1 weighted (T1w) in addition to two scans collected to estimate participant-specific field inhomogeneities. The MTw, PDw and T1w scans were all acquired using a FOV of 256 × 224 × 179 mm^3^, TR=25ms, flip angle of 6° for MTw and PDw, and 21° for T1w. The resolution was 0.8 × 0.8 × 0.8mm^3^_._ To improve image quality, i.e. maximize signal to noise ratio and minimize geometric distortion, eight gradient echoes from 2.34-18.44ms were acquired for the PDw and T1w images, and six from 2.34 – 13.84ms for the MTw image with an echo spacing of 2.30ms. B1 Transmit bias field maps were collected using a 3D EPI acquisition of spin-echo and stimulated echo images with 48 slices, TR=500ms, TE1=39.06, TE2=130ms, slice thickness=4 mm; FOV: 256  ×  192 mm^2^. The field maps were acquired with 64 slices using TR=1020ms, TE1=10ms, TE2=12.46ms, slice thickness=4 mm; FOV: 192 × 192 mm^2^. Parallel imaging acceleration was used with an acceleration factor of 2 (GRAPPA) and 3D distortion correction was applied to all images during acquisition. The total scan time for all MPM images was 24 minutes.

### 2.3 Image processing

#### 2.3.1 T1w image processing

T1w MPRAGE scans were first bias corrected using the N3 algorithm [24], then segmented into grey and white matter using the CAT12 toolbox [25] within the Statistical Parametric Mapping (SPM) software version 12 (https://www.fil.ion.ucl.ac.uk/spm/) running on MATLAB version R2012b (The Mathworks Inc, Natick, MA, USA). Within the CAT12 toolbox the ‘ultra’ processing and ‘thorough clean’ options were selected to improve segmentation accuracy. Scans were normalised using DARTEL with affine registration used for initialisation, modulated and smoothed with an 8mm smoothing kernel to allow group comparisons. All scans were visually inspected to ensure segmentation accuracy.

#### 2.3.2 Total intracranial volume (TIV)

TIV was estimated from T1 scans using the MIDAS software [26] with a protocol described by Whitwell et al. [27]. ‬‬‬‬‬‬‬‬‬‬‬‬‬‬‬‬‬‬

#### 2.3.3 Diffusion weighted image processing

DWIs were corrected for eddy- and susceptibility-induced off-resonance fields using FSL (v5.0.11) *topup* and *eddy* [28]. Diffusion tensors (DT) were fitted to DWIs using FSL *dtifit*. Participant DT volumes were aligned to a population-based DT template using linear and non-linear tensor-based registration in DTI-TK [29], [30], [31], [32], an approach previously used for studying white matter in neurodegenerative disease [19,33,34] and shown to improve TBSS [35]. DT voxel outliers were removed prior to registration.

Fractional anisotropy (FA), mean diffusivity (MD), axial diffusivity (AD) and radial diffusivity (RD) were calculated from the DTs. Neurite orientation dispersion and density imaging (NODDI) was fitted using the NODDI MATLAB toolbox with the Accelerated Microstructure Imaging via Convex Optimization (AMICO) MATLAB toolbox to output microstructure parameters of neurite density index (NDI), orientation dispersion index (ODI) and free water fraction (FWF) [36,37].

#### 2.3.4 Multiparametric map processing

Multiparametric maps are designed to measure a number of tissue properties within the brain. After processing the acquired data, there are four quantitative measures produced: PD, MT, R1 and R2*.

MPM scans were processed using the histology MRI (hMRI) toolbox version 0.2.0 [5]. They were converted to NIfTI format and preprocessing was performed to produce PD, R1, MT and R2* quantitative maps using default settings within the Statistical Parametric Mapping software (SPM version 12) in MATLAB version R2012b (The Mathworks Inc, Natick, MA, USA). RF sensitivity bias correction was calculated via the Unified Segmentation method, and B1 bias correction was performed via the RF transmit (B1+) and receive (B1-) field measurements. Quantitative maps calculated from the three multi-echo spoiled gradient echo scans were visually examined after pre-processing.

Next, the data were processed using the voxel-based quantification (VBQ) approach [38]. This approach aims to preserve the quantitative nature of the data, whilst enabling group-wise whole-brain analysis in both the grey and white matter separately. As in VBM, there are three steps: segmentation, spatial normalization and tissue-weighted smoothing. The pipeline was applied using default settings [5] with the addition of a manually segmented whole-brain mask generated as part of the volumetric image processing [19] applied to the segmented regions to improve delineation of the grey matter (GM). An 8mm smoothing kernel was used, and visual quality control performed after each step in the pipeline.

Based on the results of the VBQ analyses, additional post-hoc regression analyses of R2*, MT and volume were performed. These analyses were conducted to understand the relationship between group or CSF NfL, R2* and MT (i.e., iron and myelin) in clusters that showed either significantly increased R2* in PreHD compared to controls, or a significant relationship between R2* and CSF NfL in PreHD. Average R2* and MT values were extracted from the smoothed data masked by each of the significant clusters. To approximate grey or white matter volume within these clusters, probability values from the unsmoothed grey and white matter maps created as part of the VBQ processing were also extracted from each cluster (i.e. if the cluster was within the white matter, white matter volume was calculated). The proportional values represent how much of each region is grey or white matter, and thus are an approximation for volume; volume as measured in the MPRAGE analysis was not used for the regression analysis because it was processed in a different imaging space to the MPM analysis and was not directly comparable. Extracted values for R2*, MT and volume for each cluster were used for statistical analysis.

#### 2.3.5 G-ratio processing

Magnetisation transfer (MT) and proton density (PD) maps were generated from the hMRI toolbox [5,20] as described above. The PD map was masked with the manually segmented whole-brain regions used in the MPM processing and registered to the diffusion b=0 image using FSL *flirt* and the transformation parameters then applied to the MT map. Myelin volume fraction (MVF) was calibrated from the MT using a study-specific population-based factor of 0.261 [39]. Axonal volume fraction (AVF) was calculated as (1−MVF)(1−FWF)NDI and the g-ratio as $\sqrt{1/(1+MVF/AVF)}$ [20]. Parameter maps were transformed to the DT template using the previously determined transformation field to perform TBSS.

#### 2.3.6 Neurofilament light collection and quantification

Cerebrospinal fluid (CSF) was collected via lumbar punction undertaken between 08.30am and 10.30am, with participants fasting overnight. Collection and processing were performed after the acquisition of the MRI and standardised as previously described [19,40,41]. Samples were placed on wet ice and processed within 30 minutes of collection by centrifugation and freezing using standard kits containing polypropylene plasticware supplied by the HDClarity study (https://hdclarity.net/). All samples were stored at -80°C.

Detailed quality control was conducted on data at all stages of processing, which was performed blinded to disease status and clinical data to ensure comparability of data between groups and reduce potential bias. Exclusions following quality control are detailed in the results.

### 2.4 Statistical analysis

In total, 45 outcome measures were examined in the whole brain analyses, with an additional eight post-hoc analyses being performed.

#### 2.4.1 T1w analysis

Group comparisons to test for volumetric differences between controls and HD in both grey and white matter separately were performed using linear regression models in SPM 12, controlling for age, sex and TIV. In addition, in the PreHD group associations between volume and disease burden (N = 62) and CSF NfL (N = 58) were examined. Results were evaluated at a cluster-wise threshold of *p* < .05, corrected for family-wise error (FWE). Two masks were created, one from the average grey matter segmentations and one from the average white matter segmentations. These masks were used as explicit masks in the statistical analysis. In addition, eroded versions of these masks were used as exclusion masks when extracting the results to ensure that there was no overlap between grey and white matter.

#### 2.4.2 DTI and NODDI analysis

TBSS was implemented in FSL [42]. A mask was created from the average FA using FSL *bet* and applied to all parameter maps (erroneous voxels were removed after manual inspection). The tract skeleton was created from the average FA and subject DTI and NODDI microstructure parameter maps projected onto the skeleton.

Tract spatial statistics for each DWI microstructure parameter, testing for group differences between control and gene-carriers, and in PreHD participants correlations with DBS (N=60) and CSF NfL (N=56), were computed on the skeleton using FSL *randomise* [43]. Models included age and sex as covariates. FSL *randomise* calculated the voxel-wise *p*-value and z-score of the t-statistic using 5000 permutations. Threshold-free cluster enhancement (TFCE) was then used to produce FWE-corrected *p*-values using 5000 permutations across space and those *p* < .05 were considered significant [44].

#### 2.4.3 MPM analysis

For all MPM maps analyses were performed in both the grey and white matter separately. Group comparisons were performed using linear regression models in SPM 12, controlling for age, sex and TIV. In addition, in the PreHD group associations between each quantitative map and disease burden (N = 54), and CSF NfL were examined (N = 50). Results were evaluated at a voxel-wise threshold of *p* < .001, corrected at a cluster-wise threshold of *p* < .05FWE.

#### 2.4.4 G-ratio analysis

G-ratio analysis was performed using TBSS as above for the DTI and NODDI analysis. Tract spatial statistics for G-ratio were compared for group differences between PreHD and controls. In PreHD participants, correlations with DBS (N=55) and CSF NfL (N=51), were computed on the skeleton [43]. Models included age and sex as covariates. FWE-corrected *p*-values of *p* < .05 were considered significant [44].

#### 2.4.5 Post-hoc regression analysis

Data extracted from eight clusters were examined in the post-hoc regression analysis; two significant white matter clusters from the group comparisons, four significant grey matter clusters from the correlations with CSF NfL, and two significant white matter clusters from the correlations with CSF NfL. Regression analyses were performed in RStudio version 1.2.1335. R2* was used as the dependent variable for all analysis. For the clusters that resulted from group comparisons, group, MT, volume, age, gender, and TIV were included in the model. For the clusters that resulted from the correlation analysis, CSF NfL, MT, volume, age, gender, and TIV were included in the model. All analyses were evaluated at a p value of *p* < .05.

#### 2.4.6 Role of the funding source

The study was funded by the Wellcome Trust who had no role in study design, data collection, data analyses, interpretation or writing of the report.

## 3 Results

### 3.1 Participants

#### 3.1.1 Missing data

Of 131 participants recruited to the HD-YAS study, 123 participants (62 PreHD and 61 controls) underwent imaging for the study. Reasons for exclusion from imaging included contraindications to MRI not declared during screening such as claustrophobia. No scans failed quality control for the T1 MPRAGE analysis. For the diffusion data, one scan failed quality control, one had an artefact on the DWI and one dataset was excluded as it was acquired after DWI processing began (60 PreHD and 60 controls). MPM scans were collected on 121 participants (61 PreHD and 60 Controls) Four MPM datasets (two PreHD, two controls) were subsequently excluded due to motion, six were excluded due to processing failures (five PreHD, one control), resulting in 54 PreHD and 57 control datasets. For the g ratio analysis there were 55 PreHD and 59 control participants who had both DWI data and MT/PD maps that passed quality control for this analysis. It should be noted that the number of participants excluded from the current analysis differ from those excluded in the previous HD-YAS ROI analysis [19] due to different inclusion/exclusion criteria for motion and additional processing.

CSF collection was optional for the HD-YAS study and was available on 58 PreHD and 51 control participants.

#### 3.1.2 Demographic information

Demographic criteria for the cohort are included in Table 1. The groups were matched for age, sex and education. There was a significant difference in UHDRS TMS (t-test, p<.05), although all participants had a motor score of <5, indicating that no PreHD participants were symptomatic. In addition, for TMS the median score was 0; a score in the range of 1-5 is not specific to HD and is often seen in controls [45,46].

**Table 1:** Demographic information for the HD-YAS cohort included in the current study. Values are means (standard deviation) and range. Group comparisons were made using t tests (age, education, and NART) and a χ^2^ test (sex). NA=not applicable. NART=National Adult Reading Test, an estimate for IQ. PreHD=premanifest Huntington's disease. UHDRS=Unified Huntington's Disease Rating Scale. TIV= total intracranial volume.

		PreHD (N = 62)	Controls (N = 61)	*p* value (t-test/χ^2^ test)
Age		29.08 (5.59) 19-40	29.15 (5.50) 20-39	*p* = .95
Sex	Male	33	37	*p* = .48
	Female	29	24	
Education (years)		16.24 (2.16) 12-21	16.38 (2.24) 12-22	*p* = .73
NART		102.11 (7.34) 86-120	103.67 (8.30) 86-120	*p* = .27
UHDRS total motor score		0.48 (1.04) 0-5	0.11 (.32) 0-1	*p* = .01
Total functional capacity		13 (0) 13-13	13 (0) 13-13	-
CAG repeat length		42.18 (1.64) 39-47	NA	-
Disease burden score		189.36 (39.85) 115.50-313.50	NA	-
Estimated years to onset		23.60 (5.88) 10.02-36.13	NA	-
TIV (ml)		1496.57 (162.18) 1183.65-1805.01	1486.72 (150.74) 1189.25-1815.95	*p* = .73

### 3.2 Volumetric results

There were no significant group differences at a cluster-wise threshold of *p* < .05 FWE-corrected. There were no associations between grey matter volume and DBS or CSF NfL, or between white matter volume and DBS or CSF NfL in the PreHD group.

### 3.3 DTI and NODDI microstructure metrics

There were no significant group differences in DTI or NODDI measures at a FWE-corrected threshold of *p* <.05. There were no significant associations between DTI or NODDI measures and DBS or CSF NfL in the PreHD group.

### 3.4 Multiparametric map results

**Fig. 1:**
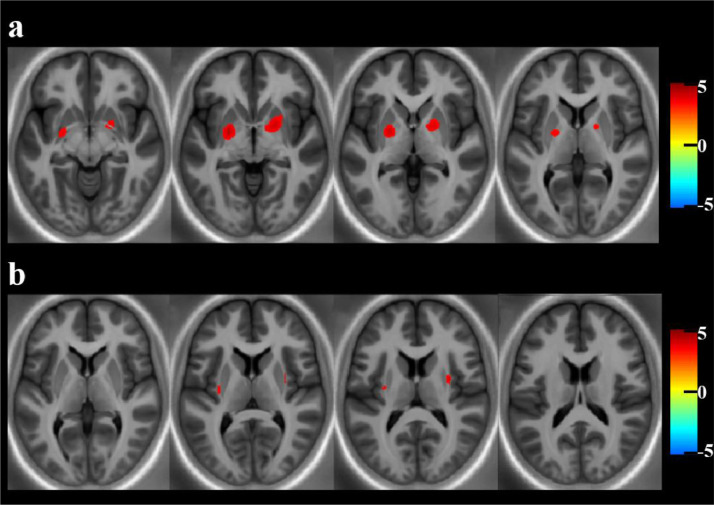
Significant results of the group comparisons between PreHD and control participants in the MPM data. a) Significantly increased R1 in the globus pallidum and putamen of the grey matter in PreHD (N = 54) compared to controls (N =57) displayed across multiple axial slices to display all clusters, and on a study-specific R2 template for visualisation purposes; b) Significantly increased R2* in the external capsule of the white matter in PreHD (N = 54) compared to controls (N =57) displayed across multiple axial slices to display all clusters, and on a study-specific R2 template for visualisation purposes. All results displayed using T-scores for clusters significant at a voxel-wise threshold of *p* < .001, corrected at a cluster-wise threshold of *p* < .05FWE and are corrected for age, sex and TIV.

There was increased R1 in the bilateral globus pallidus (GP) and putamen in PreHD compared to controls at a voxel-wise threshold of *p* < .001, corrected at a cluster-wise threshold of *p* < .05FWE (t-test), as shown in Table 2 and Fig. 1a. For R2*, there were significantly higher values in the external capsule bilaterally at a cluster-wise threshold of *p* < .05 FWE, see Table 2 and Fig. 1b. There was no evidence that MT or PD differed between controls and PreHD in white or grey matter at a voxel-wise threshold of *p* < .001, corrected at a cluster-wise threshold of *p* < .05FWE (t-test).

**Table 2:** Group comparison results for the multiparametric maps showing regions of significantly higher R1 and R2* in PreHD (N = 54) compared to controls (N = 57) (t-tests). Results are corrected at a voxel-wise threshold of *p* < .001, using a cluster-wise threshold of *p* < .05FWE.

		Hemisphere	Cluster Size	T value (cluster peak)	p_FWE – corr_	Cluster peak coordinates MNI (mm) (x, y, z)	Degrees of freedom
R1 Map	Grey matter	Left	2530	4.12	*p* < .001	-23, -5, -4	1, 106
		Right	2882	3.72	*p* < .001	21, 1, -5	
R2* Map	White matter	Right	363	3.71	*p* < .001	31, -4, 10	1, 106
		Left	183	3.36	*p* = .026	-30, -14, 8

**Fig. 2:**
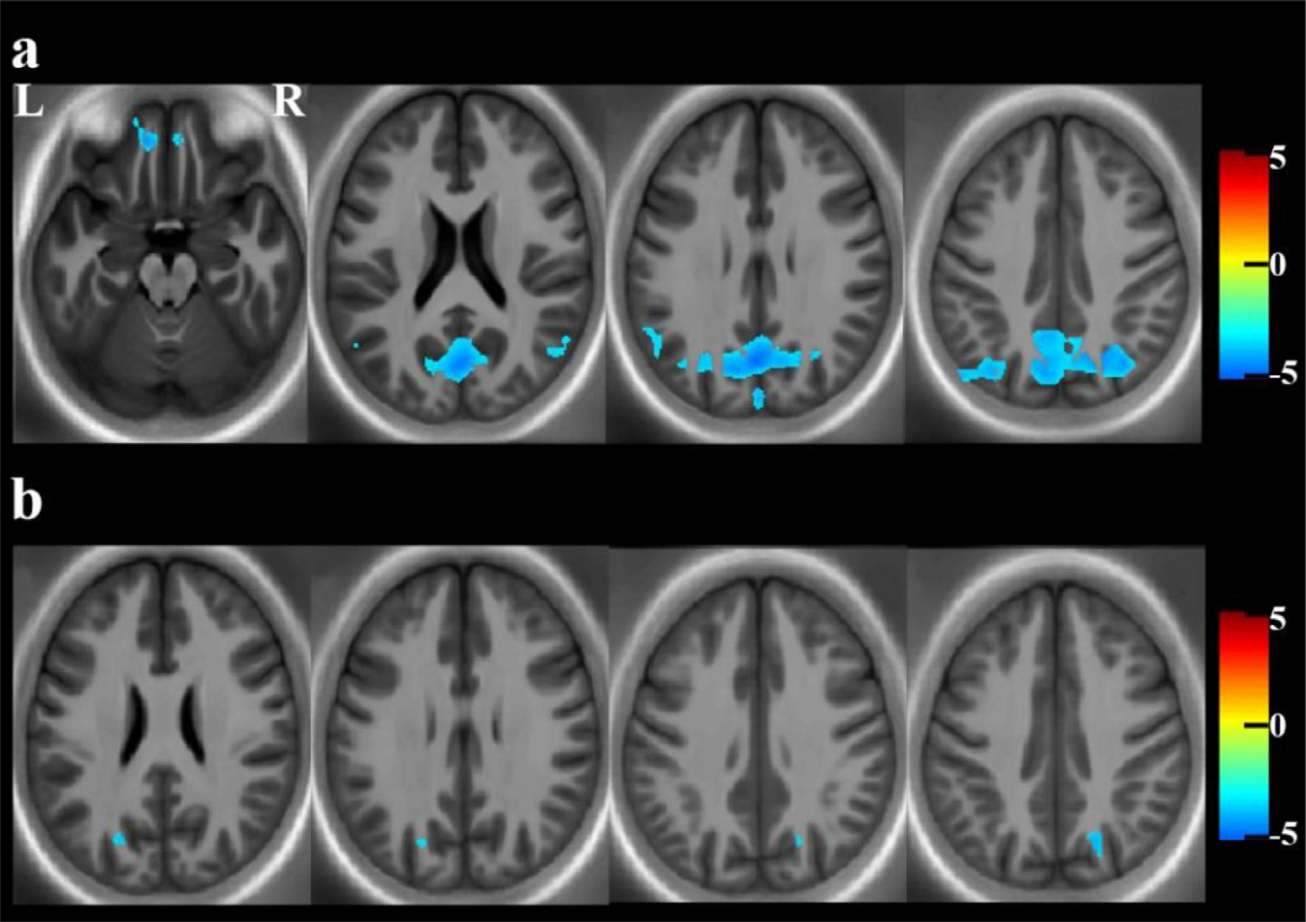
Significant results showing associations between MPM maps and CSF NfL in PreHD participants. a) Significant associations between R2* and CSF NfL in the cortical grey matter, suggestive of a relationship between higher NfL and decreased cortical myelin/iron, displayed across multiple axial slices to display all clusters, and on a study-specific R2 template for visualisation purposes; b) Significant associations between R2* and CSF NfL in the white matter, displayed across multiple axial slices to display all clusters, and on a study-specific R2 template for visualisation purposes. All results displayed using Tscores for clusters significant at a voxel-wise threshold of *p* < .001, corrected at a cluster-wise threshold of *p* < .05FWE.and are corrected for age, sex and TIV. N = 50.

**Fig. 3:**
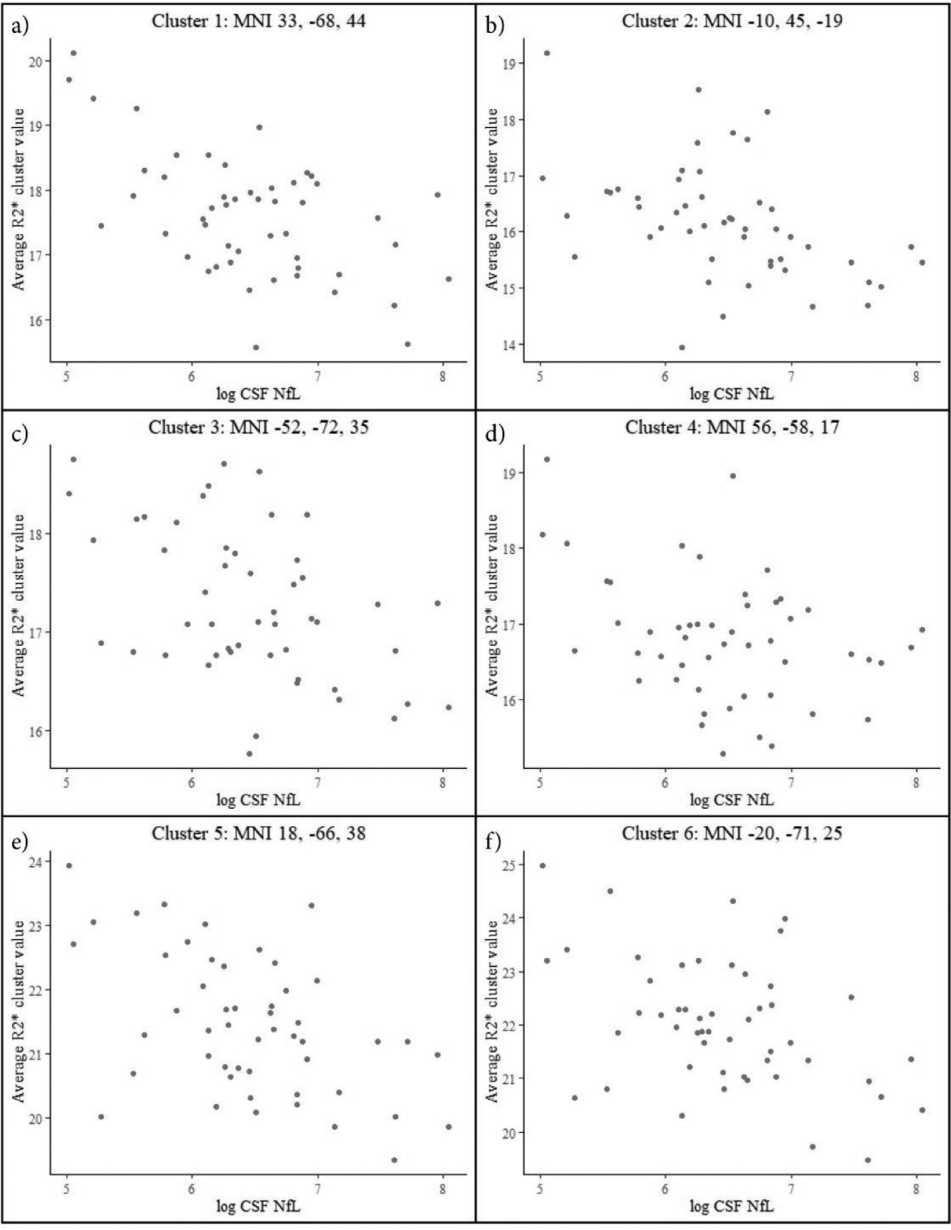
Scatterplots showing the relationship between CSF NfL and R2* in all significant clusters. For each graph the y-axis shows R2* values averaged across the cluster for each participant, and x-axis shows log CSF NfL for each participant. a) Cluster 1, MNI cluster peak coordinates: 33, -68, 44; b) Cluster 2, MNI cluster peak coordinates: -10, 45, -19; c) Cluster 3, MNI cluster peak coordinates: -34, -70, 41; d) Cluster 4, MNI cluster peak coordinates: -52, -59, 24; e) Cluster 5, MNI cluster peak coordinates: 56, -58, 17; f) Cluster 6, MNI cluster peak coordinates: 22, -72, 43; g) Cluster 7, MNI cluster peak coordinates: -21, -71, 26. Data are presented adjusted for age, sex and TIV, N = 50.

For CSF NfL, there was a significant negative relationship between R2* and CSF NfL in parieto-occiptal and frontal grey matter regions. Similarly, there was a significant negative relationship between R2* and CSF NfL in parieto-occipital white matter. These results are presented in Table 3 and Fig. 2. Fig. 3 shows the mean extracted R2* values for each cluster plotted against CSF NfL for the PreHD group. There was no relationship between CSF NfL and R1, MT or PD in the white or grey matter.

**Table 3:** Significant associations between multiparametric maps and CSF NfL, N = 50 (t-test). Results are corrected at a voxel-wise threshold of *p* < .001, using a cluster-wise threshold of *p* < .05FWE.

		Cluster number	Hemisphere	Cluster Size	T value (cluster peak)	p_FWE − corr_	Cluster peak coordinates MNI (mm) (x, y, z)	Degrees of freedom
R2* Map	Grey matter	Cluster 1	Right	455560	4.79	*p* < .001	33, -68, 44	1, 45
		Cluster 2	Left	1561	4.46	*p* < .001	-10, 45, -19	
		Cluster 3	Left	9705	4.30	*p* < .001	-52, -72, 35	
		Cluster 4	Right	1000	3.91	*p* < .001	56, -58, 17	
	White matter	Cluster 5	Right	603	3.86	*p < .001*	18, -66, 38	1, 45
		Cluster 6	Left	273	3.66	*p* = .003	-20, -71, 25

There were no significant associations between DBS and MT, PD, R1 or R2* in the white or grey matter.

### 3.5 G-ratio results

There were no significant group differences in the g-ratio between PreHD and controls at a FWE-corrected threshold of *p* < .05. There were no significant associations between g-ratio and DBS or CSF NfL in the PreHD group.

### 3.6 Post-Hoc regression analysis results

The results indicate that after controlling for volume and MT, the relationship between R2* and group remained significant (multiple regression, *p* < .001). Furthermore, after controlling for volume and MT, the relationship between R2* and CSF NfL remained significant (multiple regression, *p* < .001). Full results are presented in Supplementary tables 1-3.

## 4 Discussion

This study provides evidence that early microstructural brain changes are occurring in HD gene-carriers very far from disease onset, despite the previously demonstrated absence of clinical, cognitive or psychiatric differences [10]. There was significantly higher R1 in HD gene-carriers compared to control participants in the globus pallidum (GP) and putamen, and higher R2* in the external capsule, which we hypothesise is related to higher iron. In addition, there were significant negative associations between R2* and CSF NfL, a sensitive biofluid biomarker of neurodegeneration, indicating that as NfL increases there are reductions in either iron or myelin signal. We hypothesise that this is due to very early demyelination and an associated loss of oligodendrocytes and iron, which result in reduced R2* signal. There were no other significant group differences and in the HD gene-carriers, there were no other significant associations between any imaging measure and CSF NfL and no significant relationship between disease burden, an estimate of HD disease load, and any imaging measure.

Both iron and myelin have been implicated in HD disease processes [7,8,10], and using MPMs we are able to probe both of these features in vivo via MRI. The only group differences to survive statistical correction were higher R1 and R2* in the basal ganglia and surrounding white matter, as shown in Fig. 1 and Table 2. We saw sub-threshold increased R1 and R2* in the putamen and external capsule in our previous ROI analysis [19], however in that study we did not measure the GP, where the largest change in R1 appears to be occurring. The higher R1 values in the GP, a region known to be low in myelin content but high in iron and which undergoes iron accumulation in HD [14], suggests that these observed higher R1 values reflect iron content. Previous studies have reported increased iron in the GP in HD, but our results show that this is occurring much earlier than previously thought [14,16]. Although higher R2* in the external capsule has not been reported previously in HD, the higher R2* seen here could be occurring as oligodendrocytes attempt to maintain the pyramidal neurons that have heavily myelinated axons. The lack of association between any of our measures in these regions and CSF NfL, a marker of axonal degeneration shown to be elevated in the HD-YAS cohort, suggests that degeneration of the axons extending from these regions is not yet occurring. Alternatively iron could be accumulating in both the GP and external capsule regions independently due to changes in iron homeostasis resulting from the effects of the mutant Huntingtin protein.

The lack of significant findings in MT maps, which are sensitive to myelin, supports our conclusion that the results seen here are related to iron accumulation, rather than myelin. Furthermore, our post-hoc analysis was designed to interrogate the relationship between R2* and MT, i.e. iron and myelin. After controlling for MT (myelin) and volume, the relationships between R2* (iron) and group, and R2* (iron) and CSF NfL remained significant. These results support our conclusion that the group differences in R2* and correlations between R2* and CSF NfL are likely to be occurring due to changes in iron rather than myelin.

Bilateral regions of the posterior parietal cortex and precuneus, along with a small region of the left frontal cortex, showed a significant negative association between R2* and CSF NfL. In our recent study [19], we showed that CSF NfL was elevated in this very far from onset cohort and not associated with changes in striatal volume, the results from this study show that the early increase in CSF NfL is linked to microstructural changes in the posterior parietal-occipital cortex, a region previously shown to undergo some of the earliest cortical changes in HD [4,47,48]. R2* can reflect both iron and myelin content [49], with changes in iron and myelin both occurring during axonal degeneration [50]. The negative association indicates that lower R2* is associated with higher NfL, which we hypothesise is due to a loss of oligodendrocytes and associated iron resulting from very early demyelination. Similar findings have been shown previously in both white and cortical grey matter, particularly in multiple sclerosis [51], [52], [53]. Although we did not see significant congruent changes in other maps thought to represent myelin qualities (particularly MT maps), discrepancies between the detection and location of R2* and MT signal have been reported previously in MPMs, possibly due to differences in cortical architecture and thus MRI signal [54] and very early changes could result in reduced iron due to the loss of oligodendrocytes, prior to myelin degredation.

It is important to note that although the results found in this analysis are suggestive of disease-related processes in HD many years prior to HD symptom onset, the lack of specificity in each MPM metric limits the interpretation of these results and we cannot definitively determine the origin of the signal in R1 and R2* maps. Our post-hoc analysis attempted to corroborate our conclusion that the signal change in R2* is driven by iron rather than myelin. We also saw no results in the MT maps indicative of alterations in myelin, however is important to note that R2* signal is influenced by myelin (although to a lesser degree than iron), and so we cannot definitively conclude that the R2* results are driven by iron changes alone. Further imaging and pre-clinical work focussed on myelin and iron in HD will help to elucidate the true origin of the signal change seen here.

Using ROI techniques we previously found evidence of decreased putamen volume along with a non-significant decrease in caudate volume in this cohort. Although we did not detect significantly reduced volume of these structures here, this might be accounted for by the reduced power in a whole-brain VBM analysis. In the current study, at an uncorrected threshold we saw regions of reduced volume in the putamen and caudate in the PreHD group compared to the control group, supporting our previous findings that there are very subtle reductions in subcortical volume.

The lack of diffusion-weighted imaging findings in this study is in agreement with the findings of our ROI analysis and a connectivity-based analysis also performed in this cohort (Zeun et al., under review), and suggest that even when using a state-of-the-art diffusion acquisition in a large cohort, there are no consistent detectable changes in measures of white matter integrity this many years prior to disease onset. Furthermore, the lack of findings in the g-ratio analyses indicate that there are no significant changes in axonal myelin sheath thickness. Given the importance of the myelin sheath in nerve signal conduction, these results might indicate that neuronal communication is not affected this far from onset in PreHD. Whilst we did detect some significant findings in the white matter of the MPMs, these were not extensive, and together our results suggest that the white matter is mostly preserved at this point in premanifest disease.

We saw a discrepancy between the group comparisons (higher R1/R2* in and around the subcortex) and the correlations with CSF NfL (negative correlations between R2* and CSF NfL in the parieto-occipital cortices and the frontal lobe). The group differences were detected in iron rich subcortical regions previously shown to accumulate iron in HD, and thus have been interpreted as an accumulation of iron rather than myelin changes. These results were not related to either DBS or NfL. This is consistent with volumetric results reported in this cohort previously, whereby we saw reduced volume in the putamen in PreHD which was not associated with markers of disease load [19]. There is increasing evidence that neurodevelopment is affected in HD [55,56] and it is possible that these results represent developmental elements of HD not associated with ongoing neurodegeneration, however it is important to note that this study was not designed to detect developmental changes due to HD and further longitudinal follow up work would needed to be done to test this theory. In contrast, while there were associations between NfL and R2* in cortical brain regions, there were no significant group differences in these regions. This suggests that brain changes in this region are subtle and thus on a group level they do not differ from control participants.

There were no significant associations between DBS and any imaging measures. DBS is an estimated measure of disease load calculated from age and CAG repeat and thus does not represent ongoing disease processes as well as CSF NfL, a direct measure of axonal health which has been shown as a highly sensitive biomarker in HD and which varies across the disease course, particularly in PreHD [57]. CAG repeat-length does not fully account for individual variance seen in disease onset and progression, and thus disease burden incorporates this measurement error [58]. In contrast, CSF NfL is thought to act as a dynamic marker of HD disease progression which closely tracks disease progression [57]. The difference between these measures may result in the incongruity between the analyses.

It is important to recognise limitations to this study. The whole-brain nature of these analyses mean that statistical power is reduced compared to ROI methods, however we wanted to undertake exploratory analyses to complement our previous ROI study. Indeed, despite the reduced power we saw significant results in both the group differences and correlation analyses in regions not studied via the ROI analysis. To date there are no studies using such extensive microstructural measures in HD; it would be important to perform studies using similar extensive microstructural measures in late PreHD and early manifest HD participants in comparison to HD-YAS to understand the trajectory of iron and myelin changes across the course of the disease, which we expect will vary as disease processes change [51]. Finally, we limited our correlation analyses to two markers of disease stage and progression – one estimated and one measured. We chose not to perform associations with any cognitive, psychiatric measures as there was no evidence of disease related change in these measures in our cohort. Instead, we selected DBS due to its standing as a commonly used estimate of disease load across the full spectrum of HD gene-carriers and CSF NfL due to the significant effect found in the HD-YAS cohort [19]. For this study we recruited PreHD and control participants from across the UK via a range of HD clinics, genetic clinics and support groups. We attempted to recruit participants in order that the results are widely generalizable, however these results require replication in other datasets.

Here, we undertook a comprehensive analysis examining macro- and micro-structure of a number of MRI measures in HD gene-carriers approximately 24 years from disease onset, including the first application of MPMs. Our results indicate that in the basal ganglia and associated white matter, the iron accumulation previously reported in later stages of HD has already begun. In posterior parietal and occipital regions known to undergo early changes in HD we see a significant negative association between R2* and CSF NfL, interpreted as reduced myelin with higher levels of CSF NfL. Our results suggest that the early rise in CSF NfL in HD gene-carriers is related to early degenerative processes taking place in the cortex which lead to a reduction in regional iron and myelin levels. This analysis demonstrates that microstructural changes can be detected via MRI in PreHD participants many years prior to disease onset, providing further evidence that cortical iron and myelin play an important role in HD pathogenesis.

## Declaration of Competing Interest

SJT reports grants from Wellcome Trust, grants from UK Dementia Research Institute, during the conduct of the study; other from F. Hoffmann La Roche Ltd, personal fees from F. Hoffmann La Roche Ltd, personal fees from Annexon, personal fees from PTC Therapeutics, personal fees from Takeda Pharmaceuticals Ltd, personal fees from Vertex Pharmaceuticals Incorporated., personal fees from UCB Pharma S.A, personal fees from Alnylam Pharmaceuticals Inc, personal fees from Decision Resources Group, personal fees from Genentech, personal fees from DDF Discovery Ltd, personal fees from Triplet Theraputics, personal fees from Novartis, outside the submitted work. GR reports grants from Wellcome Trust during the conduct of the study. All other authors declare no competing interests.

## Appendix List of HD-YAS investigators

Dr Claire O'Callaghan

Prof Ed Wild

Dr Cristina Sampaio

Prof Trevor Robbins

Prof Barbara Sahakian

Dr Oliver Quarrel

Dr Nayana Lahiri

Dr Andrea Nemeth

Dr Mary Porteous

Dr Elisabeth Rosser

Dr David Craufurd

Dr Rhona MacLeod

Dr Deborah Ruddy

Dr Roger Barker

Dr Simon Holden

Dr Hugh Rickards

Dr Anne Rosser

Dr Emma Hobson

Prof Angus Clarke

Dr Katherine Lachlan

Dr Reza Kiani

Dr Timothy Harrower

## Appendix Supplementary materials

Image, application 1

Image, application 2
