# Supplementary material for: Altered iron and myelin in premanifest Huntington's Disease more than 20 years before clinical onset: Evidence from the cross-sectional HD Young Adult Study
Source: eBioMedicine. 2021 Mar 9;65:103266. doi: 10.1016/j.ebiom.2021.103266 (PMC7960938; doi:10.1016/j.ebiom.2021.103266)
Supplement: Supplementary file 1 [file mmc1.docx]

**Supplementary information**

Altered iron and myelin in premanifest Huntington’s Disease more than 20 years before clinical onset: Evidence from the cross-sectional HD Young Adult Study

**Supplementary Figure 1: Example maps for all DTI, NODDI, G-ratio and MPM metrics.**


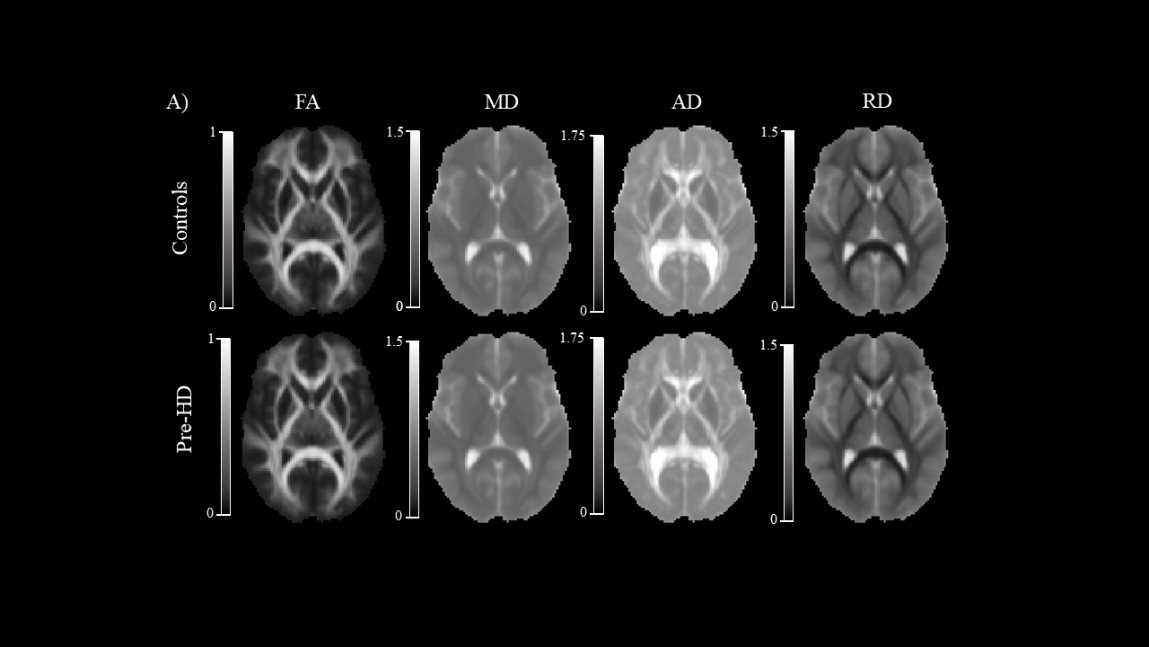

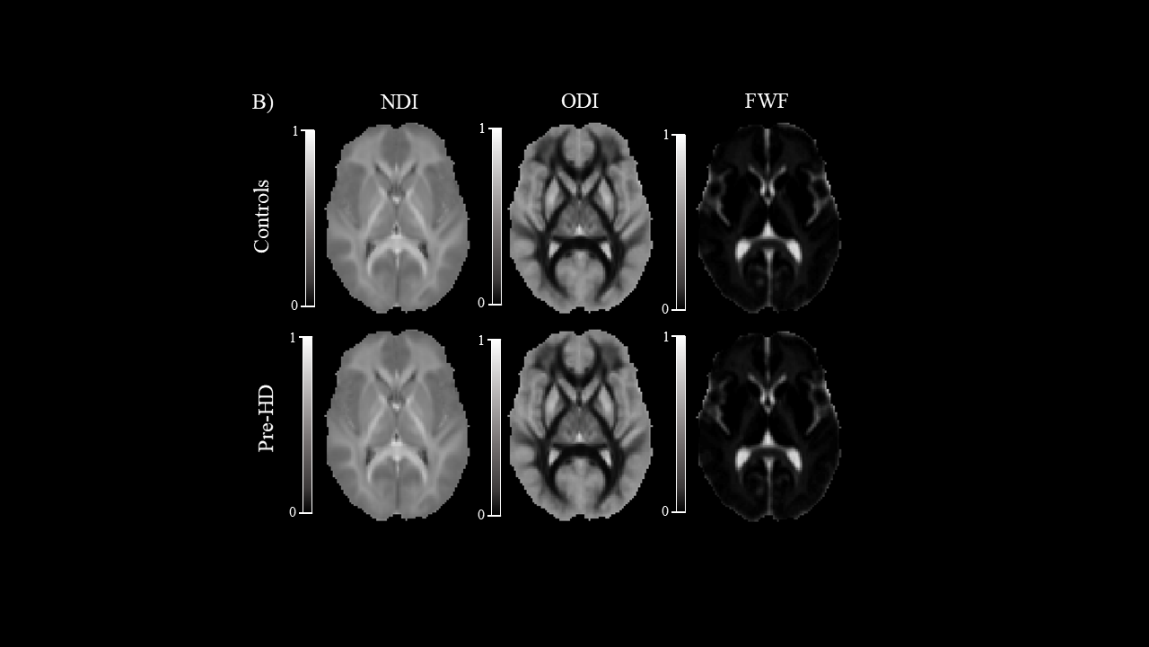

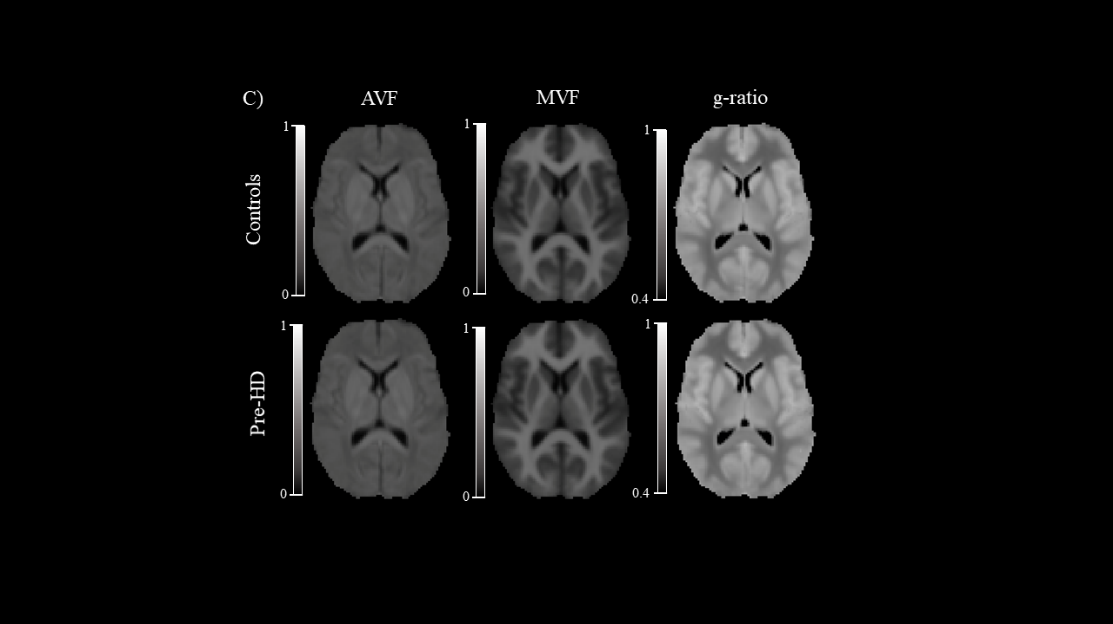

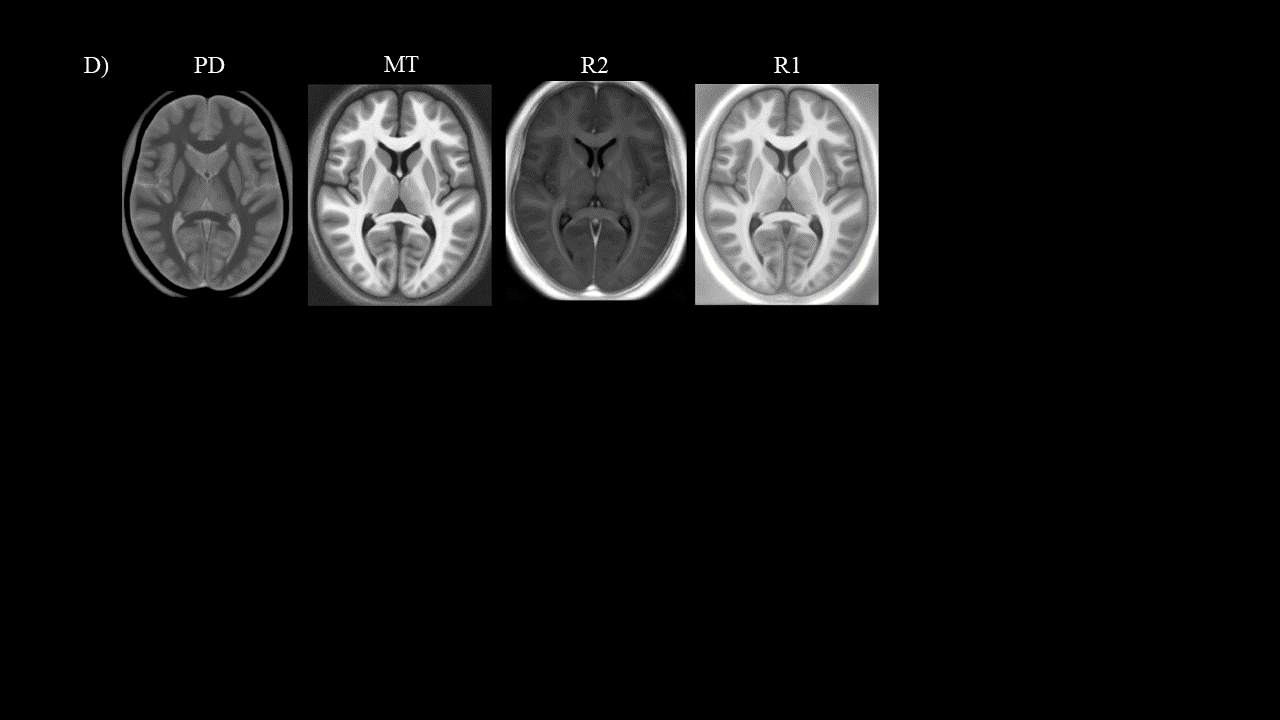


Figure 1: The images in Figure 1 display sample templates for all imaging types analysed in the study, with axial slices presented for each modality to give an understanding for visualization purposes. A) Shows DTI metrics for a study-specific template scan (fractional anisotropy (FA), mean diffusivity (MD), axial diffusivity (AD), radial diffusivity (RD)) for controls and pre-HD participants; B) Shows average NODDI metrics for a study-specific template scan (neurite density index (NDI), orientation dispersion index (ODI) and free water fraction (FWF)) for controls and pre-HD participants; C) Shows average g-ratio metrics for a study-specific template scan (axonal volume fraction (AVF), myelin volume fraction (MVF), g-ratio) for controls and pre-HD participants; D) shows average MPM metrics for controls and pre-HD participants in a study-specific template scan (proton density (PD), magnetization transfer (MT), effective transverse relaxation rate (R2), longitudinal relaxation (R1).

|  | **Clusters from the group comparison:**  **White matter**  N = 111 | |
| --- | --- | --- |
|  | **Cluster 1**  R^2^ = 0.27, *p < .001****  DF = 104 | **Cluster 2**  R^2^ = 0.18, *p = .002***  DF = 104 |
| Group | 0.58 (0.16)  *p* < 0.001*** | 0.67 (0.19)  *p* < 0.001*** |
| MT | 5.98 (1.74)  *p* < 0.001*** | 2.56 (1.90)  *p* = .011** |
| Volume | -6.30 (3.04)  *p* = 0.040* | -1.85 (2.96)  *p* > 0.050 |
| Age | 0.03 (0.01)  *p* = .041* | 0.04 (0.02)  *p* > 0.050 |
| TIV | 1. (0.00)   *p* > 0.050 | 1. (0.00)   *p* > 0.050 |
| Gender | -0.22 (0.23)  *p* > 0.050 | -0.03 (0.28)  *p* > 0.050 |

Supplementary Table 1: Results of analyses predicting R2* value from group, magnetization transfer (MT), volume, age, total intracranial volume (TIV) and gender for two clusters in the white matter. Clusters were determined based on the results of the whole-brain VBQ analysis. The table shows R^2^, *p* value and degrees of freedom (DF) for each model, with the beta estimate, standard error (in parenthesis) and *p* value shown for each predictor. N = 111, * *p* < .05, ** *p* < .01, *** *p* < .001.

|  | **Clusters from the correlation analyses:**  **Grey matter**  N = 50 | | | |
| --- | --- | --- | --- | --- |
|  | **Cluster 1**  R^2^ = 0.48, *p < .001****  DF = 43 | **Cluster 2**  R^2^ = 0.10, *p = .425*  DF = 43 | **Cluster 3**  R^2^ = 0.43, *p < .001****  DF = 43 | **Cluster 4**  R^2^ = 0.45, *p < .001****  DF = 43 |
| CSF NfL | -0.99 (0.17)  *p* < 0.001*** | -1.02 (0.20)  *p* < 0.001*** | -0.76 (0.16)  *p* < 0.001*** | -0.71 (0.16)  *p* < 0.001*** |
| MT | 4.98 (4.01)  *p* > 0.050 | 2.91 (6.10)  *p* > 0.050 | 4.39 (3.23)  *p* > 0.050 | 4.39 (3.16)  *p* > 0.050 |
| Volume | -5.61 (4.89)  *p* > 0.050 | 0.39 (4.32)  *p* > 0.050 | 1.84 (3.05)  *p* > 0.050 | 1.46 (1.65)  *p* > 0.050 |
| Age | 0.05 (0.02)  *p* > 0.050 | 0.10 (0.03)  *p* < 0.010** | 0.06 (0.02)  *p* < 0.001*** | 0.09 (0.02)  *p* < 0.001*** |
| TIV | 0.00 (0.00)  *p* > 0.050 | 0.00 (0.00)  *p* > 0.050 | 0.00 (0.00)  *p* > 0.050 | 0.00 (0.00)  *p* > 0.050 |
| Gender | 0.50 (0.37)  *p* > 0.050 | 0.67 (0.40)  *p* > 0.050 | 0.30 (0.31)  *p* > 0.050 | 0.24 (0.32)  *p* > 0.050 |

Supplementary Table 2: Results of analyses predicting R2* value from CSF NfL, magnetization transfer (MT), volume, age, total intracranial volume (TIV) and gender for four clusters in the grey matter. Clusters were determined based on the results of the whole-brain VBQ analysis. The table shows R^2^, *p* value and degrees of freedom (DF) for each model, with the beta estimate, standard error (in parenthesis) and *p* value shown for each predictor. N = 50, * *p* < .05, ** *p* < .01, *** *p* < .001.

|  | **Clusters from the correlation analyses:**  **White matter**  N = 50 | |
| --- | --- | --- |
|  | **Cluster 1**  R^2^ = 0.54, *p < .001****  DF = 43 | **Cluster 2**  R^2^ = 0.35, *p = .003***  DF = 43 |
| CSF NfL | -0.84 (0.18)  *p* < 0.001*** | -0.89 (0.24)  *p* < 0.001*** |
| MT | 7.51 (1.70)  *p* < 0.001*** | 5.80 (2.21)  *p* < 0.050* |
| Volume | -2.50 (0.86)  *p* < 0.010** | 0.02 (2.55)  *p* > 0.050 |
| Age | 0.01 (0.02)  *p* > 0.050 | 0.05 (0.03)  *p* > 0.050 |
| TIV | 0.00 (0.00)  *p* > 0.050 | 0.00 (0.00)  *p* > 0.050 |
| Gender | 1.04 (0.38)  *p* < 0.010** | 0.53 (0.50)  *p* > 0.050 |

Supplementary Table 3: Results of analyses predicting R2* value from CSF NfL, magnetization transfer (MT), volume, age, total intracranial volume (TIV) and gender for two clusters in the white matter. Clusters were determined based on the results of the whole-brain VBQ analysis. The table shows R^2^, *p* value and degrees of freedom (DF) for each model, with the beta estimate, standard error (in parenthesis) and *p* value shown for each predictor. N = 50, * *p* < .05, ** *p* < .01, *** *p* < .001.
