# Supplementary material for: Altered iron and myelin in premanifest Huntington's Disease more than 20 years before clinical onset: Evidence from the cross-sectional HD Young Adult Study
Source: eBioMedicine. 2021 Mar 9;65:103266. doi: 10.1016/j.ebiom.2021.103266 (PMC7960938; doi:10.1016/j.ebiom.2021.103266)
Supplement: Supplementary file 2 [file mmc2.doc]

STROBE Statement—Checklist of items that should be included in reports of ***cross-sectional studies***

|  | Item No | Recommendation |
| --- | --- | --- |
| **Title and abstract** | 1 | (*a*) Indicate the study’s design with a commonly used term in the title or the abstract – added ‘cross-sectional’ to title |
|  |  | (*b*) Provide in the abstract an informative and balanced summary of what was done and what was found  - completed |
| Introduction | | |
| Background/rationale | 2 | Explain the scientific background and rationale for the investigation being reported  - completed |
| Objectives | 3 | State specific objectives, including any prespecified hypotheses - hypothesis clearly stated in introduction and final paragraph of introduction provides specific objectives |
| Methods | | |
| Study design | 4 | Present key elements of study design early in the paper  -study design outlined in final paragraph of the introduction with further detail in the participant section of the methods |
| Setting | 5 | Describe the setting, locations, and relevant dates, including periods of recruitment, exposure, follow-up, and data collection  -included in participant information |
| Participants | 6 | (*a*) Give the eligibility criteria, and the sources and methods of selection of participants  -included in participant information |
| Variables | 7 | Clearly define all outcomes, exposures, predictors, potential confounders, and effect modifiers. Give diagnostic criteria, if applicable -information on imaging outcomes provided in image analysis section. Diagnostic criteria not applicable as participants were premanifest. |
| Data sources/ measurement | 8* | For each variable of interest, give sources of data and details of methods of assessment (measurement). Describe comparability of assessment methods if there is more than one group  -information provided in imaging methods and statement declaring comparability of data included at the end of this section |
| Bias | 9 | Describe any efforts to address potential sources of bias  -included a statement that analysis was performed blind to disease status and clinical data to reduce potential bias |
| Study size | 10 | Explain how the study size was arrived at  -information included in participant section |
| Quantitative variables | 11 | Explain how quantitative variables were handled in the analyses. If applicable, describe which groupings were chosen and why - included in the statistical analysis section |
| Statistical methods | 12 | (*a*) Describe all statistical methods, including those used to control for confounding -included in statistical analysis section |
|  |  | (*b*) Describe any methods used to examine subgroups and interactions N/A |
|  |  | (*c*) Explain how missing data were addressed  - missing data section included at the beginning of the results |
|  |  | (*d*) If applicable, describe analytical methods taking account of sampling strategy N/A |
|  |  | (*e*) Describe any sensitivity analyses N/A |
| Results | | |
| Participants | 13* | (a) Report numbers of individuals at each stage of study—eg numbers potentially eligible, examined for eligibility, confirmed eligible, included in the study, completing follow-up, and analysed  - included at the start of the results |
|  |  | (b) Give reasons for non-participation at each stage  - completed |
|  |  | (c) Consider use of a flow diagram  - due to shortage of space and relative simplicity of the data collection we don’t feel this is necessary |
| Descriptive data | 14* | (a) Give characteristics of study participants (eg demographic, clinical, social) and information on exposures and potential confounders  - included in results and Table 1 |
|  |  | (b) Indicate number of participants with missing data for each variable of interest  - included at the beginning of results section |
| Outcome data | 15* | Report numbers of outcome events or summary measures  - This is now given in statistical analysis section. |
| Main results | 16 | (*a*) Give unadjusted estimates and, if applicable, confounder-adjusted estimates and their precision (eg, 95% confidence interval). Make clear which confounders were adjusted for and why they were included  N/A for MRI imaging study |
|  |  | (*b*) Report category boundaries when continuous variables were categorized  N/A |
|  |  | (*c*) If relevant, consider translating estimates of relative risk into absolute risk for a meaningful time period  N/A |
| Other analyses | 17 | Report other analyses done—eg analyses of subgroups and interactions, and sensitivity analyses  N/A |
| Discussion | | |
| Key results | 18 | Summarise key results with reference to study objectives  -provided in opening sentence of the discussion |
| Limitations | 19 | Discuss limitations of the study, taking into account sources of potential bias or imprecision. Discuss both direction and magnitude of any potential bias  -limitations discussed on page 24 |
| Interpretation | 20 | Give a cautious overall interpretation of results considering objectives, limitations, multiplicity of analyses, results from similar studies, and other relevant evidence  -completed |
| Generalisability | 21 | Discuss the generalisability (external validity) of the study results - discussed on page 24 |
| Other information | | |
| Funding | 22 | Give the source of funding and the role of the funders for the present study and, if applicable, for the original study on which the present article is based  -completed on page 26 |

*Give information separately for exposed and unexposed groups.

**Note:** An Explanation and Elaboration article discusses each checklist item and gives methodological background and published examples of transparent reporting. The STROBE checklist is best used in conjunction with this article (freely available on the Web sites of PLoS Medicine at http://www.plosmedicine.org/, Annals of Internal Medicine at http://www.annals.org/, and Epidemiology at http://www.epidem.com/). Information on the STROBE Initiative is available at www.strobe-statement.org.
